# The Effect of Chronic Prostatitis/Chronic Pelvic Pain Syndrome (CP/CPPS) on Semen Parameters in Human Males: A Systematic Review and Meta-Analysis

**DOI:** 10.1371/journal.pone.0094991

**Published:** 2014-04-17

**Authors:** Weihua Fu, Zhansong Zhou, Shijian Liu, Qianwei Li, Jiwei Yao, Weibing Li, Junan Yan

**Affiliations:** 1 Department of Urology, Southwest Hospital, Third Military Medical University, Chongqing, People's Republic of China; 2 Institute for Pediatric Translational Medicine, Shanghai Children's Medical Center, Shanghai Jiaotong University School of Medicine, Shanghai, People's Republic of China; Northwestern University, United States of America

## Abstract

**Background:**

Chronic prostatitis/chronic pelvic pain syndrome (CP/CPPS) is one of the risk factors of impaired male fertility potential. Studies have investigated the effect of CP/CPPS on several semen parameters but have shown inconsistent results. Hence, we performed a systematic literature review and meta-analysis to assess the association between CP/CPPS and basic semen parameters in adult men.

**Methods:**

Systematic literature searches were conducted with PubMed, EMBASE and the Cochrane Library up to August 2013 for case-control studies that involved the impact of CP/CPSS on semen parameters. Meta-analysis was performed with Review Manager and Stata software. Standard mean differences (SMD) of semen parameters were identified with 95% confidence intervals (95% CI) in a random effects model.

**Results:**

Twelve studies were identified, including 999 cases of CP/CPPS and 455 controls. Our results illustrated that the sperm concentration and the percentage of progressively motile sperm and morphologically normal sperm from patients with CP/CPPS were significantly lower than controls (SMD (95% CI) −14.12 (−21.69, −6.63), −5.94 (−8.63, −3.25) and −8.26 (−11.83, −4.66), respectively). However, semen volume in the CP/CPPS group was higher than in the control group (SMD (95% CI) 0.50 (0.11, 0.89)). There was no significant effect of CP/CPPS on the total sperm count, sperm total motility, and sperm vitality.

**Conclusions:**

The present study illustrates that there was a significant negative effect of CP/CPPS on sperm concentration, sperm progressive motility, and normal sperm morphology. Further studies with larger sample sizes are needed to better illuminate the negative impact of CP/CPPS on semen parameters.

## Introduction

Prostatitis is a common male urogenital disease with prevalence ranging from 2.2% to 9.7% worldwide, with an overall rate of 8.2% [1]. A heterogeneous mixture of syndromes defines prostatitis, with broad diagnostic criteria and a vague understanding of its etiology and pathophysiology [2], [3]. To improve its clinical diagnosis, the National Institutes of Health (NIH) classifies prostatitis into four categories, namely, I: acute bacterial prostatitis, II: chronic bacterial prostatitis, III: chronic prostatitis/chronic pelvic pain syndrome (CP/CPPS), and IV: asymptomatic inflammatory prostatitis [4].

CP/CPPS accounts for more than 90% of all symptomatic prostatitis cases in urology outpatient clinics [4]. It is characterized by chronic pelvic pain symptoms, which lasted at least 3 months during the previous 6 months, in the absence of a urinary tract bacterial infection but in the presence of urinary symptoms and sexual dysfunction [4], [5]. These symptoms seriously affect the quality of life of patients [6], [7]. Based on the presence or absence of leukocytes in prostatic secretions (EPS), postprostatic massage urine (VB3), or semen, CP/CPPS is further subdivided into two subtypes: NIH IIIA (inflammatory) and NIH IIIB (noninflammatory) [4], [8]. Traditionally, symptomatic prostatitis without bacteriuria was defined as nonbacterial prostatitis (inflammatory) or prostatodynia (noninflammatory) on the basis of leukocytes in EPS [9]. Compared with the traditional EPS-based classification, NIH IIIA encompasses a larger range of patients than nonbacterial prostatitis due to its broader criteria for inflammation. In other words, patients diagnosed with prostatodynia may be categorized into the NIH IIIB or NIH IIIA subgroup [10], [11].

During the past decade, the incidence of male accessory gland infection (MAGI) as a potential etiologic factor in male subfertility or infertility has increased [12], [13]. Adverse factors, including pathogenic bacteria, leukocytes, cytokines, reactive oxygen species (ROS), obstruction and immunological allergic effects, might be involved in the development of infertility resulting from MAGI [13], [14]. As one of the main clinical categories of MAGI, chronic prostatitis can also affect male fertility [14]–[16]. For example, *Chlamydia trachomatis* infection is related to poor semen quality in prostatitis patients [17]–[19]. However, as for the negative effect of CP/CPPS on semen quality, published studies present conflicting results, with some studies showing statistically significant alterations of basic semen parameters due to CP/CPPS [20]–[23], but not others [24], [25]. Therefore, we systematically reviewed the available literature and performed a meta-analysis to evaluate the association between CP/CPPS and basic semen parameters in adult men, which might shed valuable insights on the treatment of infertility.

## Methods

### Literature search

This meta-analysis was restricted to published studies that investigated the effect of CP/CPPS on semen parameters during male reproductive age. Two independent reviewers (Li QW and Yao JW) searched PubMed, EMBASE, and the Cochrane Library from inception to August 2013, without restrictions on language or study type. The search terms combined text words and MeSH terms. For example, the search terms for CP/CPPS were prostatitis, prostatism, chronic prostatitis, chronic pelvic pain syndrome, abacterial prostatitis, nonbacterial prostatitis, and prostatodynia, while those for semen parameters were semen, sperm, spermatozoa, spermatozoon, semen analysis, sperm count, spermatozoon count, sperm motility, spermatozoon motility, and spermatozoon density. All related articles and abstracts were retrieved. In addition, references cited within relevant reviews were retrieved by hand.

### Eligibility criteria

#### Inclusion criteria

Studies were included if patients met diagnostic criteria for CP/CPPS according to the NIH classification or the traditional definition of nonbacterial prostatitis and prostatodynia. The controls were healthy human males. Semen samples were obtained before therapeutic intervention and analyzed according to World Health Organization (WHO) criteria. Semen parameters included seminal plasma volume, sperm concentration (density), total sperm count, motility, vitality and morphology. Available data were extracted from the article, including means and standard deviations of sperm parameters in all case-control groups.

#### Exclusion criteria

Studies were excluded if they were case reports. Studies involving patients with chronic prostatitis accompanied by other disorders of the urogenital system, patients that had previously undergone surgery of the genital system, and patients previously diagnosed with azoospermia or infertility were excluded. Studies involving patients with a mean age of <12 years old or >60 years old were also excluded [26].

### Study selection and validity assessment

Two independent reviewers (Li QW and Yao JW) screened titles and abstracts of all citations from the literature search. All relevant studies that appeared to meet eligibility criteria were retrieved. Full texts were needed to analyze if an ambiguous decision was made based on the title and abstract. The final decision of eligible studies was made by reviewing articles. Disagreements were resolved by consensus or a third reviewer (Zhou ZS). Two reviewers (Liu SJ and Zhou ZS) completed the quality assessment according to the primary criteria for nonrandomized and observational studies of the Newcastle-Ottawa Quality Assessment scale (NOS) in meta-analyses [27].

### Data extraction and statistical analysis

Data, including demographic data (authors, year of publication, country, number and mean age of participants, and abstinence time) and outcome data of semen parameters (semen volume, sperm concentration, total sperm count, sperm progressive motility, sperm total motility, sperm vitality, and sperm normal morphology), were extracted from the studies by three reviewers (Li QW, Yao JW and Zhou ZS). Disagreements were resolved by consensus.

Quantitative meta-analysis was performed by two reviewers (Liu SJ and Fu WH) with Review Manager (RevMan) software (version 5.2.5, The Nordic Cochrane Centre, The Cochrane Collaboration, 2012, Copenhagen) and Stata software (version 12.0, College Station, Texas, USA). Semen parameter data were analyzed in the meta-analysis. To better understand the effect of CP/CPPS on semen parameters, patients were classified into three subgroups according to the NIH classification: NIH IIIA, NIH IIIB or NIH III (the subgroup of unclassified CP/CPPS) in our study. We pooled the standard mean differences (SMD) of semen parameters of the case-control groups, which were identified with 95% confidence intervals (95% CI). Heterogeneity was assessed by the P-value and the I-square statistic (I^2^) in the pooled analyses, which represents the percentage of total variation across studies [28]. If the P-value was less than 0.1 or the I^2^-value was greater than 50%, the summary estimate was analyzed in a random-effects model. Otherwise, a fixed-effects model was applied. In addition, publication bias was detected by visual symmetry of funnel plots, with asymmetry suggesting possible publication bias. It was also assessed by the Begg's and Egger's test in the meta-analysis. If the P-value was less than 0.05, publication bias existed.

## Results

### Characteristics of the included studies

**Figure 1:**
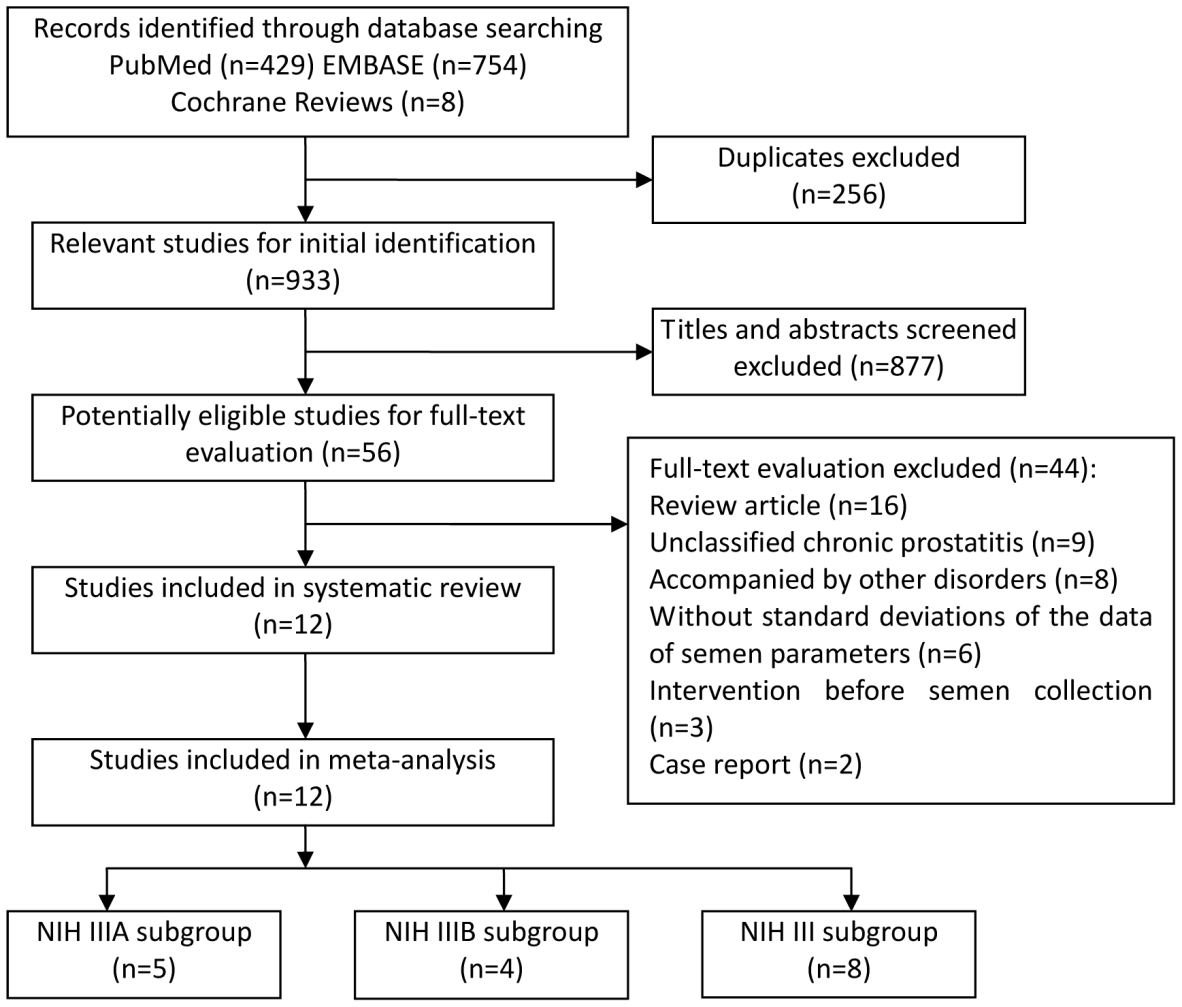
Flow diagram of selection of eligible studies.

Figure 1 shows a detailed review process. A total of 933 nonduplicate studies were identified. Twelve studies were ultimately selected according to eligibility criteria (Table 1). Of these, five [25], [29]–[32], four [29], [31], [33], [34], and eight [25], [29], [32], [35]–[39] studies investigated the effects of NIH IIIA, NIH IIIB, and NIH III (unclassified CP/CPPS) on semen parameters, respectively. All retrieved studies involved 999 CP/CPPS cases and 455 controls.

**Table 1:** Characteristics of included studies investigating the effect of CP/CPPS on semen parameters.

Study	Country	Mean age (case/control)	Abstinence time (days)	IIIA (n)	IIIB (n)	III (n)	Control (n)	Semen parameters	Criterion for semen analysis
Ausmees *et al*. 2013	Estonia	55.3/56.1	5.9±3.8			213	35	SV, TSC, SC, SPM, SNM	1999/2010 WHO manual
Zhao *et al*. 2008	China	30.3/28.9	3–7			60	20	SV, SC, SPM, SpV	1999^a^ WHO manual
Motrich *et al*. 2006	Argentina	40.41/32.18	2–7			25	15	SV, SC, SNM, SPM, SpV	1999 WHO manual
Wang *et al*. 2006	China	35/32	5–7		74		46	SV, SC, SPM, SpV	1999^a^ WHO manual
Henkel *et al*. 2006	Germany	40.4–37.3/33.2	NI	24	32	56	95	SC, SPM, SpV, STM, SNM	1999 WHO manual
Motrich *et al*. 2005	Argentina	41.41/32.18	2–7			29	15	SC, SV, SPM, SpV, STM, SNM	1999/1992 WHO manual
Zhang *et al*. 2004	China	27.5/NI	3–7	86			20	SV, SC, SPM, SNM	1999^a^ WHO manual
Menkveld *et al*. 2003	Germany	NI/NI	NI	34	18		17	SV, SC, SPM, STM, SpV, SNM	1999/1992 WHO manual
Engeler *et al*. 2003	Switzerland	41/42	NI		30		30	SV, SC, STM, SNM	1999 WHO manual
Pasqualotto *et al*. 2000	USA	NI/NI	>2	5		39	19	SC, SPM, SNM	1999 WHO manual
Leib *et al*. 1994	Israel	39.5/31.4	4			86	101	SV, TSC, SpV, SPM, SNM	1992 WHO manual
Weidner *et al*. 1991	Germany	41.2/39.5	<7	102		142	42	SC, SPM	1987 WHO manual

Abbreviations: SpV, semen volume; SC, sperm concentration (density); TSC, total sperm count; SPM, progressive sperm motility; STM, total sperm motility; SpV, sperm vitality; SNM, normal sperm morphology; IIIA, NIH IIIA subgroup; IIIB, NIH IIIB subgroup; III, NIH III subgroup; NI, not indicated in studies;

a : confirmed by the authors.

Table 1 summarizes general data from the twelve studies. The mean ages of patient and control groups were in the ranges of 27.5–55.3 years and 28.9–56.1 years, respectively. The mean ages of patient and control groups were unavailable for three studies [30]–[32]. All but one of these studies reported exclusion/inclusion criteria [31]. Nine out of twelve studies included the abstinence time before semen collection [25], [30], [32], [33], [35]–[39]. Semen analysis was performed according to WHO criteria. Two studies [31], [37] also evaluated sperm morphology according to stringent criteria described by Menkveld and Kruger [40], [41], in order to comparing with the results assessed by the 1992 WHO manual. These data were not included in this meta-analysis.

### Meta-analysis

**Figure 2:**
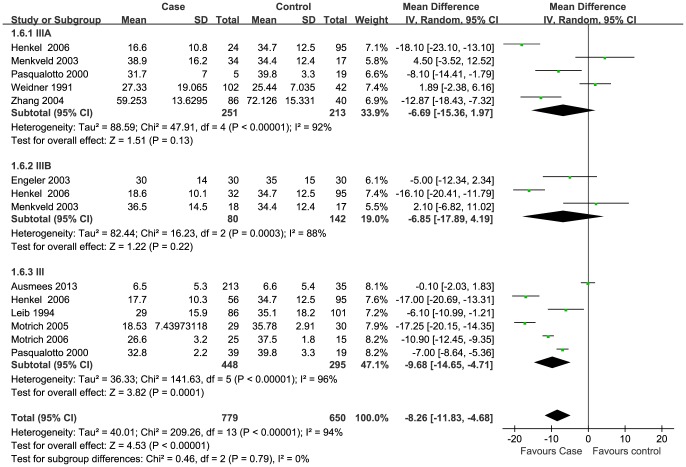
Forest plot showing the meta-analysis outcomes of the effect of CP/CPPS on sperm normal morphology.

**Figure 3:**
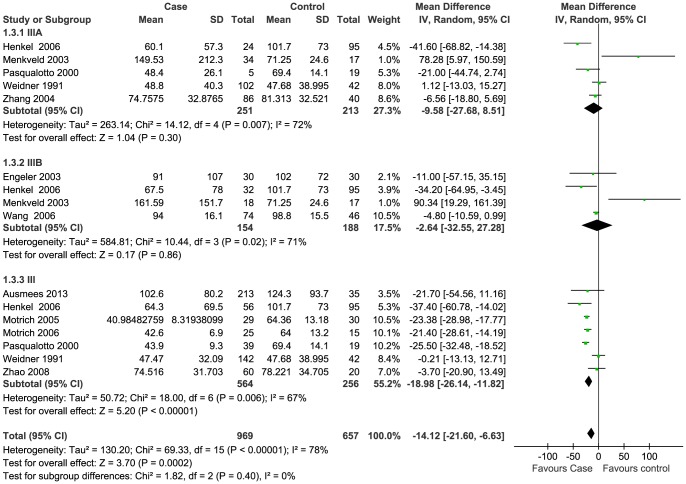
Forest plot showing the meta-analysis outcomes of the effect of CP/CPPS on sperm concentration.

**Figure 4:**
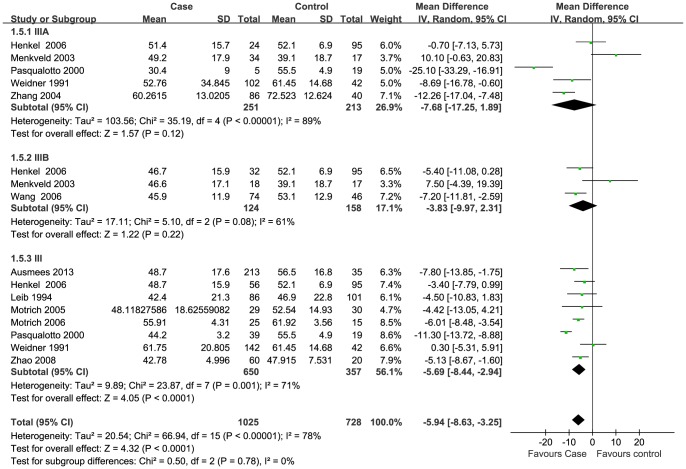
Forest plot showing the meta-analysis outcomes of the effect of CP/CPPS on sperm progressive motility.

**Figure 5:**
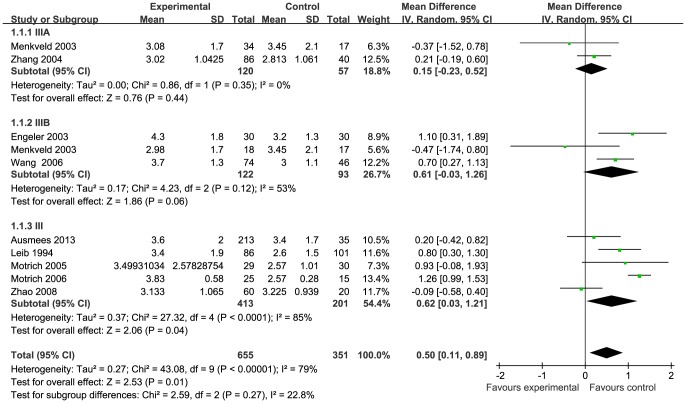
Forest plot showing the meta-analysis outcomes of the effect of CP/CPPS on semen volume.

**Figure 6:**
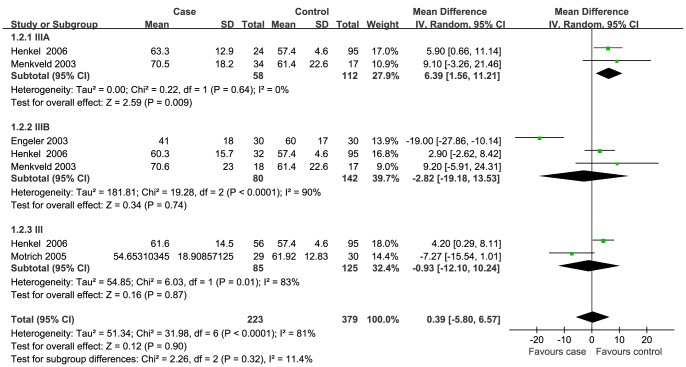
Forest plot showing the meta-analysis outcomes of the effect of CP/CPPS on sperm total motility.

**Figure 7:**
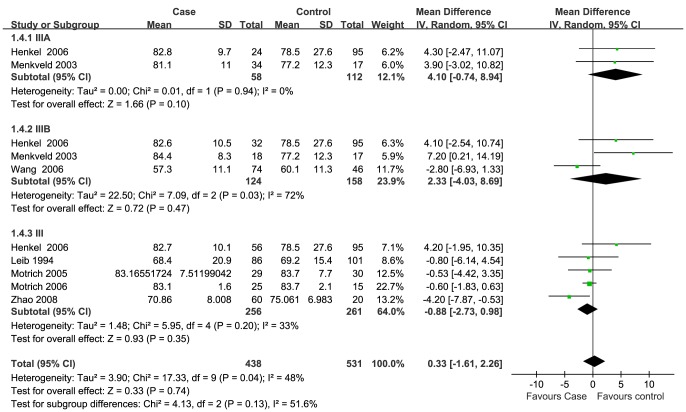
Forest plot showing the meta-analysis outcomes of the effect of CP/CPPS on sperm vitality.

**Figure 8:**
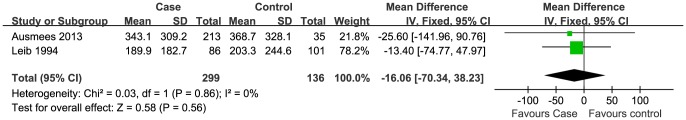
Forest plot showing the meta-analysis outcomes of the effect of CP/CPPS on total sperm counts.

Data of seven semen parameters were respectively analyzed in a random-effects model to estimate the effect of CP/CPPS on each parameter. The results suggested that sperm concentration and the percentage of progressively motile sperm and morphologically normal sperm from patients with CP/CPPS were significantly lower than controls. Pooled SMD (95% CI) were −14.12 (−21.69, −6.63), −5.94 (−8.63, −3.25), and −8.26 (−11.83, −4.66), respectively. There was evidence of significant heterogeneity among these studies (P<0.01, I^2^>75%) (Figures 2, 3, 4). Semen volume was higher in the CP/CPPS group than in the control group (SMD (95% CI): 0.50 (0.11, 0.89)). There was also evidence of significant heterogeneity among these studies (P<0.000001, I^2^ = 79%) (Figure 5). However, there was no effect of CP/CPPS on sperm total motility, sperm vitality, and total sperm count in the meta-analysis (Figures 6, 7, 8).

Subgroup analysis was performed simultaneously. There was a statistically significant difference in the same four semen parameters between the NIH III subgroups and the control groups. Pooled SMD and 95% CI for semen volume, sperm concentration, sperm progressive motility, and normal sperm morphology were 0.62 (0.03, 1.21), −18.98 (−26.14, −11.82), −5.69 (−8.44, −2.94), and −9.68 (−14.65, −4.71), respectively. There was evidence of significant heterogeneity among these studies (P<0.01, I^2^>50%) (Figures 2, 3, 4). However, there was no significant difference in any of the semen parameters according to subgroup analysis of the NIH IIIA and the NIH IIIB, except for the percentage of sperm total motility in the NIH IIIA subgroup (SMD (95% CI): 6.39 (1.56, 11.21)). There was no evidence of significant heterogeneity among these studies (P = 0.64, I^2^ = 0%) (Figure 6).

## Discussion

In this study, twelve available published articles were reviewed and analyzed statistically to investigate the effect of CP/CPPS on seven semen parameters. The results of this meta-analysis suggested that CP/CPPS significantly reduced sperm concentration, sperm progressive motility, the percentage of normal sperm morphology, and increased semen volume of patients compared with controls. The relationship between CP/CPPS and total sperm count, sperm total motility, and sperm vitality was not identified.

Basic semen parameters are still the mainstay of male fertility and reproductive health assessment [42], [43]. For example, the percentage of morphologically normal sperm is an important indicator of male fertility potential and testicular stress [44]. Poor sperm morphology characterized by poor chromatin condensation, acrosome reaction, or DNA integrity is related to sperm dysfunction [45]. In our review, nine studies investigated the effect of CP/CPPS on normal sperm morphology. Positive results were shown in five studies [29], [30], [37]–[39], but not in the remaining four studies [31], [32], [34], [35]. Our results illustrate that CP/CPPS associated with a significant decline in the percentage of morphologically normal sperm (Figure 2), which is consistent with a previous literature review [12].

It is noteworthy that there is a downward trend in the percentage of morphologically normal sperm with the published time of the included studies, especially the very low percentage (6.0% for controls) reported by Ausmees et al. [35]. Besides negative environmental and socio-psycho-behavioral factors [46], [47], the heterogeneity of normal sperm morphology is mainly due to the implementation of strict criteria and additional criteria for sperm morphology evaluation [47]. Evaluation criteria of sperm morphology has passed through two phases, liberal and strict criteria [48]. In the five consecutive editions of the WHO laboratory manual, liberal criteria were adopted in the 1980 and 1987 manuals [49], [50], and strict (Tygerberg) criteria were accepted in part in the 1992 manual [51] and recommended in the 1999 and 2010 manuals [43], [52]. Owing to the increasingly stringent criteria, the cut-off reference values for normal sperm morphology were significantly reduced from 80.5% in the 1980 WHO manual to 4% in the 2010 WHO manual [43], [49].

Some studies have argued that very low normal sperm morphology evaluated with strict criteria limits its clinical value in investigating male fertility potential [53]–[55]. To make up for this deficiency, additional sperm morphology parameters such as abnormal sperm morphology, acrosome index (AI), teratozoospermia index (TZI), and sperm pattern defects have been studied [47]. In the included studies of this review, the percentages of sperm head, midpiece, and tail defects were higher in CP/CPPS patients than in controls [29], [31], [39]. However, statistically significant differences in morphologically abnormal sperm, AI and TZI were not found between case-control groups [25], [31], [33].

Sperm concentration is an important indicator of semen quality. An enhanced level of DNA damage was observed in semen samples with low sperm concentrations [56]. However, there is no linear relationship between sperm concentration and fecundity. Previous studies reported a decrease in male fertility when the sperm concentration was below the threshold value, the range of which is 15×10^6^/ml to 55×10^6^/ml [57]–[60]. In the most recent WHO manual, a normal sperm concentration is 15×10^6^/ml [43]. However, sperm concentration is dependent on semen volume to some extent. Therefore, total sperm count might be a better indicator of normal spermatogenesis. It was not only positively correlated with testis size [61], but also with the time interval from wish of pregnancy to pregnancy obtained [62].

In this review, the sperm concentration was measured in eleven out of twelve studies [25], [29]–[38]. Statistically significant differences were found between case and control groups in only two studies [31], [38], in which the patients were respectively diagnosed with NIH IIIB and chronic nonbacterial prostatitis with positive lymphoproliferative autoimmune response against prostate antigens. The pooled SMD result suggested a statistically significant decrease in sperm concentration in the CP/CPPS group (Figure 3). However, owing to the significant increase in semen volume in patients with CP/CPPS, a statistically significant difference in total sperm count was not found in the CP/CPPS group compared to the control group in our meta-analysis (Figure 8). It should point out that the data of total sperm count was shown in only two included studies [35], [39]. Other two included articles also involved it, but without the exact values in texts [29], [32]. Contradictory conclusions were drawn in these four studies, and only Henkel *et al.* reasoned that total sperm counts were significantly reduced in NIH IIIA and NIH IIIB groups compared to control groups (P<0.01) [29].

Sperm motility is essential for fertilization. Impaired sperm motility is significantly associated with unstable DNA/RNA and mitochondrial dysfunction [63]–[66], which reduce the successful fertilization rate. Therefore, the percentage of motile sperm is a good indicator of male fertility potential [67], [68]. To identify the subfertile males, the 2010 WHO manual defines the reference limit for total motility at 40% and progressive motility at 32%, and males with sperm motility values below these threshold values are asthenozoospermic [43]. Similar threshold values were reported in a previous review [59].

Infection and inflammation of the male genitourinary tract is detrimental to sperm motility. Biological and biochemical changes in seminal plasma such as the presence of leukocytes, ROS and inflammatory cytokines can impair sperm motility and fertility potential [69]. A recent review also concluded that there might be a negative impact of CP/CPPS on sperm motility [12]. In this review, all included studies revealed that sperm motility was decreased by different degrees in men with CP/CPPS, except for one study with contradictory results [31]. According to the meta-analysis, the percentage of progressive motile sperm was significantly lower in the CP/CPPS group than in the control group (Figure 4), but an adverse effect on motile total sperm was not found.

Sperm vitality is defined as the percentage of live spermatozoa [43]. It can be used to differentiate between necrozoospermia and total asthenozoospermia, and evaluate cellular membrane integrity and abnormal flagella [70]. In our review, seven studies reported conflicting sperm vitality results [29], [31], [33], [36]–[39]. Only Zhao et al. reported a significant decrease of sperm vitality in CP/CPPS patients compared with controls (P<0.05) [36]. However, in this meta-analysis, the association between CP/CPPS and semen vitality was not identified (Figure 7).

Subgroup analysis was also performed in our study. In the meta-analysis of two subgroups (NIH IIIA and NIH IIIB). The results illustrated little effect of the two CP/CPPS subtypes on seven semen parameters, except for a significant increase in sperm total motility in the NIH IIIA subgroup, which are inconsistent with the total outcomes of the meta-analysis. The reasons for the conflicting results may be that there are not sufficient valid data for the meta-analysis of the two subgroups, since only five studies involving 251 NIH IIIA patients and four studies involving 154 NIH IIIB patients were included. Moreover, not all semen parameters were investigated in individual studies. In addition, data of semen parameters were inconsistent among the only few studies.

Although the relationship between CP/CPPS and male infertility has always been controversial, a voluminous literature suggests that CP/CPPS may negatively affect sperm parameters in many ways, including seminal oxidative stress, inflammatory cytokines and autoimmune responses. Excessive ROS and lower total anti-oxidant capacity (TAC) was found in all patients with CP/CPPS compared with normal controls [32]. The increased seminal oxidative stress correlates with impaired sperm motility which was mentioned above, furthermore, it induces chromatin cross-linking, DNA strand breaks and peroxide-mediated sperm plasma membrane damage, which accelerates apoptosis and affects normal sperm morphology [71], [72]. In addition, the oxidative stress decreases acrosin activity and sperm-oocyte fusion capability [72].

Cytokines play a key role in the inflammatory response. Some inflammatory cytokines in seminal plasma of patients with CP/CPPS are increased significantly, such as IL-1, IL-6, IL-8, IL-10 and TNF-alpha, when compared with the normal group [73], [74]. Lampiao et al. reported that seminal IL-6 significantly reduced sperm progressive motility, which was possibly due to overproduction of nitric oxide (NO) [75]. Similarly, Kopa Z and colleagues thought that seminal plasma IL-6 correlated negatively with sperm vitality and sperm motility [76]. Several studies investigated the effect of TNF-alpha on sperm parameters suggest that seminal plasma TNF-alpha adversely affects sperm motility and normal morphology via impairing sperm mitochondrial function, increasing NO production, inducing apoptosis [13], [75]. As for the role of seminal IL-8, some research suggested it negatively correlated with total sperm count and sperm progressive motility [77], [78]. Review the previous studies, the relationships between IL-1/10 and semen parameters remain unclear and need further investigation [13].

Another explanation for the alterations observed in semen parameters of patients with CP/CPPS is that autoimmune responses against prostate antigens may affect semen quality, including prostatic acid phosphatase (PAP), prostate steroid-binding protein (PSBP), prostate specific antigen (PSA) and other antigens in prostate homogenates and seminal plasma [79]–[81]. The autoimmune responses are considered to reduce sperm motility, counts, normal morphology and viability, and NO, ROS, TNF-alpha, IFN-gamma and other inflammatory mediators may be involved in the impact on semen parameters [38], [81], [82].

### Limitations

There are some limitations in our study, which need to be taken into consideration when interpreting the results of this meta-analysis. First, there were differences among participants and methods, including differences in age and geographic locations of participants, different durations of abstinence before semen collection, and different editions of the WHO manual for semen analysis, all of which may have affected the results of semen analysis [46], [83]–[85]. Second, the sample size of each study was relatively small, and a total of 999 CP/CPPS patients and 455 controls were investigated in all twelve studies. Furthermore, several studies related to the subject were excluded due to lack of control data or data presented by mean ± SD [20]–[24].

### Conclusions

This systematic review and meta-analysis supports the negative effects of CP/CPPS on semen parameters. The sperm concentration and percentage of progressively motile sperm and morphologically normal sperm were significantly lower in patients with CP/CPPS. In addition, semen volume was higher in the CP/CPPS group. Multicenter clinical trials with larger sample sizes are needed to validate these findings. Further studies that focus on differences in the two subtypes of CP/CPPS may also improve our understanding of CP/CPPS on semen parameters.

## Supporting Information

Checklist S1 **PRISMA checklist.**
(DOC)
